# The Moral Regime of Midwifery Continuity of Carer Implementation in England

**DOI:** 10.1111/1467-9566.70087

**Published:** 2025-09-24

**Authors:** Aimee Louise Middlemiss, Susan Channon, Julia Sanders, Heather Strange, Rebecca Milton, Sara Kenyon, Tina Prendeville, Susan Barry, Aled Jones

**Affiliations:** ^1^ School of Nursing and Midwifery University of Plymouth Plymouth UK; ^2^ Centre for Trials Research Cardiff University Cardiff UK; ^3^ School of Healthcare Sciences Cardiff University Cardiff UK; ^4^ School of Health Sciences University of Birmingham Birmingham UK; ^5^ Women's Health Research Centre Imperial College London & Imperial College NHS Trust London UK; ^6^ Division of Women's Children's and Clinical Support Imperial College Healthcare NHS Trust London UK

## Abstract

Since 2016, the National Health Service (NHS) in England has been aiming to implement midwifery continuity of carer (MCoC), a model of maternity care in which the same midwife or small group of midwives provides antenatal, intrapartum and postnatal care for women, other birthing people and their babies. However, the implementation has faced significant difficulties. As part of a wider investigation into barriers to and facilitators of MCoC implementation, we carried out qualitative interviews with senior stakeholders involved in the implementation of MCoC at regional or national level in England. In this paper, we present an analysis of sociocultural values in the accounts of these stakeholders. We find these expert accounts of MCoC implementation are underpinned by a ‘moral regime’ which privileges certain norms and practices whilst deterring others. These accounts produce an idealised midwife‐subject who is passionate and evangelical about MCoC as a form of care and seeks to persuade others to their cause, including through the use of evidence. We conclude with some thoughts about the range of possible consequences produced by this moral regime regarding the implementation of MCoC as a maternity policy in the English NHS, and the role of moral regimes in healthcare politics.

## 1 Introduction

Since 2016, the NHS in England has prioritised the implementation of a model of maternity care known as midwifery continuity of carer (MCoC). MCoC is a way of organising midwifery care which aims for a woman and her baby^1^ to be cared for throughout pregnancy, the intrapartum period and the postnatal period by the same midwife, or small team of midwives (Homer et al. 2019). It frequently involves the use of on‐call systems to facilitate midwife availability during labour and birth (Middlemiss, Channon, et al. 2024). MCoC is supported by the World Health Organisation (WHO 2017) and has been implemented to different extents in several countries including Australia (Maternity Services Review 2009) and New Zealand (Grigg and Tracy 2013). Attempts to improve continuity within NHS midwifery care had been in existence since the 1980s (McIntosh 2012), and in the 1990s formed part of a national review of maternity care, Changing Childbirth, led by Baroness Cumberlege (Department of Health 1993). However, implementation stalled and a new review of maternity care, led again by Baroness Cumberlege, was commissioned in 2015 after serious maternity care and culture failures were identified at Morecambe Bay NHS Trust (Kirkup 2015; Department of Health 2015). The 2016 report, Better Births, revitalised MCoC (NHS England 2016), proposing increased safety and a better service would be achieved through the reorganisation of the midwifery way of working rather than additional investment in maternity services, and centring midwives as the primarily agents of change (Madsen 2025).

Better Births, and the guidance issued in support of its implementation, set ambitious targets for MCoC including intrapartum care to be the default model of midwifery organisation across England (NHS 2021; NHS England 2016, 2017). This second attempt to introduce MCoC was bolstered at the time by an influential Cochrane review which found MCoC to be associated with improved clinical outcomes, including significant reductions in preterm birth and foetal and neonatal deaths (Sandall et al. 2016). These findings were not sustained in an updated review, though MCoC was found to be associated with lower proportions of surgical births and higher maternal satisfaction with care (Sandall et al. 2024). MCoC's advantages over other models of care are considered to come from relational continuity and the building of trust between specific midwives and the women they care for, as well as informational and other forms of continuity (Haggerty et al. 2003; Saultz 2003).

The implementation of MCoC became a key priority for NHS England and was integrated into wider government and NHS strategies for maternity improvement (Department of Health and Social Care (DHSC) 2022; NHS 2019) until a new review of maternity services at the Shrewsbury and Telford NHS Trust identified yet more failures in maternity care (Ockenden 2022). The Ockenden report recommended that the national rollout of the MCoC model should be suspended until minimum staffing requirements were met, in the context of a depleted midwifery workforce in the NHS. In response, NHS England suspended targets for existing provision and further rollout of MCoC where trusts could not meet ‘minimum safe staffing requirements’ (NHS England 2022). Many trusts then scaled back provision of Better Births MCoC, although some have focused on targeted forms for specific communities such as Black and minority ethnic groups who experience severely disproportionate levels of poor maternity care (Draper et al. 2023; Peter and Wheeler 2022).

This paper forms part of a wider investigation of the attempted implementation of Better Births MCoC in England which investigates structural, systemic, institutional and processual aspects of the implementation (Milton et al. 2025). Here we analyse sociocultural values regarding MCoC expressed during qualitative interviews with national and regional stakeholders involved with, or interested in, the implementation of MCoC. Health systems have been shown to be underpinned by moral frameworks (Bartosch et al. 2024), and research studies have highlighted the role of culture, values, truth claims and power in understanding intersections of evidence and policy (Boaz et al. 2019), including during attempts at service improvement in NHS contexts (Dixon‐Woods et al. 2013). The need to consider the social and organisational context of policy in relation to the use of evidence has been noted (Redman et al. 2015), along with the selection of evidence's persuasive effect in policy contexts (Cairney 2019). We build on these insights in the specific context of MCoC implementation using a theoretical framework connecting individual subjects, discourse and possibilities of action in a ‘moral regime’ inscribed in the attempt to implement the MCoC policy in the English NHS.

### 1.1 Regimes of Truth and Moral Regimes

The concept of a moral regime is a development of Foucault's concept of regimes of truth (Foucault 1980, 1998), a feature of social and political analysis located in governmentality. Regimes of truth concern what is counted as a truth, who is able to produce and verify truth and the consequences of speaking a ‘truth’ (Foucault 2003b; Rose 1999). In this analysis, truth is not an external reality but something made, through political relations and action, in specific circumstances. It accumulates through permitted, promoted, disseminated discourse, allowing for, and structuring, further discursive truths and actions. A moral regime is similar, but the appeal to and definition of discursive authority and power is not through truth but through classificatory ideas of good and bad, and of value. Discursive parameters come about through a moral authority which is diffuse and situated in discourse and ideas, rather than a hierarchical or institutional form of control. A moral regime both constrains and produces possibilities of action and individual subjectivities (Foucault 2003a).

Moral regimes have been identified in policies, including stratified maternity provision (Castro and Savage 2019), reproductive policy (Morgan and Roberts 2012), maternity employment policy (Middlemiss, Boncori, et al. 2024), health promotion (Mathar and Jansen 2010) and in education (Qvarsebo 2019), where moral regimes have been differentiated from regimes of truth by situating them in ethical rather than epistemological politics (Qvarsebo 2019). By contrast, in this paper we argue that in the context of MCoC implementation, moral regimes and regimes of truth are intertwined and integral to possible action in health policy implementation. In line with other research (Cowan et al. 2022), we show that knowledge—in our case, ‘the evidence supporting MCoC implementation’—is intimately connected to ideas of morality.

By describing how the moral regime of MCoC implementation is constituted through an analysis of the discourse of stakeholders involved in the change process, we contribute to understandings of the implementation of change. Although implementation frameworks commonly used in health research, such as the Consolidated Framework for Implementation research (CFIR) (Damschroder et al. 2022) address aspects of individual motivation and sociocultural values, they do not describe the integration of processes of truth production and dissemination with ideas of morality and value. This paper extends the implementation science focus which considers whether an evidence base is ‘robust’ (Damschroder et al. 2022) to consider evidence use and presentation in active practice. It builds on research on policy use of evidence (Redman et al. 2015) and evaluation (Weiss et al. 2016) concerning how evidence can be deployed—for example, instrumentally, politically, or tactically. Our research study proposes a theoretical basis for connections between evidence and values, and between personal and professional motivation, in the context of implementation.

## 2 Methods

Institutional ethical approval was obtained from the University of Plymouth (reference 3906) and authors A.M., S.C., A.J. and H.S. conducted qualitative interviews with participants. These authors are non‐midwife researchers with expertise in health services research, sociology and anthropology. None had prior experience of MCoC or held a position on its desirability. Our team also includes practicing and academic midwives with experience of working in or alongside MCoC models who discussed emerging findings in regular research team meetings, and periodic Project Advisory Group (PAG) meetings discussed findings and interpretation. PAG members included experienced health services researchers, senior academic and policy midwives, midwives with experience of MCoC implementation, and Patient and Public Involvement and Engagement (PPIE) representatives. Findings were therefore repeatedly tested, discussed and challenged throughout the research project by stakeholders with diverse professional and intellectual perspectives and expertise.

**FIGURE 1:**
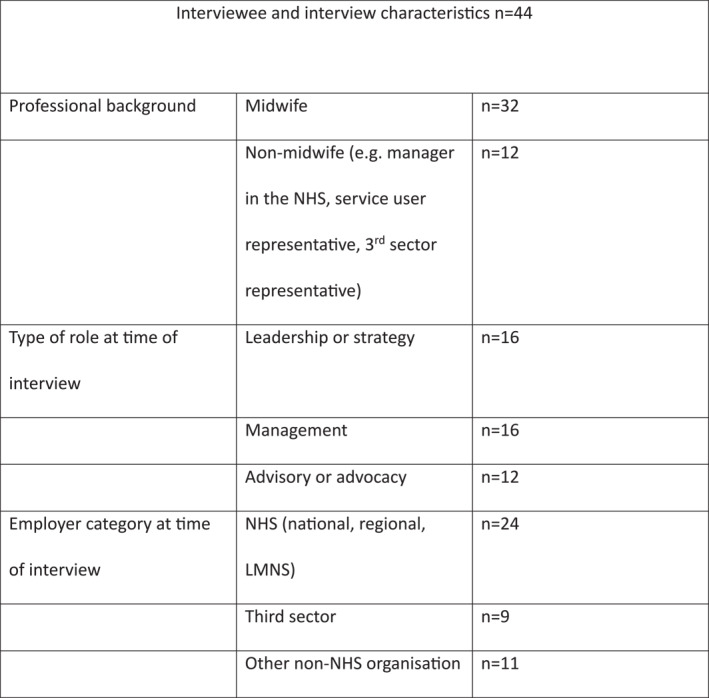
Characteristics of national and regional interviewees and interviews.

Purposive sampling identified national and regional stakeholders involved in, or with an interest in, MCoC implementation and policy during and since Better Births (NHS England 2016). These were targeted through knowledge within the wider research team and the participants' current or former roles in key NHS institutions, professional bodies, third sector and service user organisations and through ‘snowball’ sampling. We did not recruit based on prior knowledge of participants' personal positions on the desirability of MCoC. For ethical reasons we do not detail the roles of participants—some general characteristics can be found in Figure 1. Data adequacy in terms of new themes in the data and the range of interviewees covered was a factor towards the end of interviewing. Besides the 44 interviews reported here, we requested a further 54 interviews which did not take place. Thirty‐six individuals and institutions did not respond to requests, 12 refused (of which eight said they were not the relevant person or did not have time) and six interviews did not happen for logistics reasons. Absent from the sample as potential stakeholders were national or regional obstetricians, who did not respond. This may be in part due to our more limited networks with obstetricians and is a limitation of the research. The research was also limited by the accessibility of NHS organisations (e.g., Local Maternity and Neonatal Systems [LMNS] and Integrated Care Boards [ICB] do not always list contact details or respond to email). Some organisations selected one participant to represent their position, and this may have excluded dissenting voices or alternative narratives. Role changes during the period of MCoC and the timescale of the implementation may have meant some information might be less current. Furthermore, those who responded may have had particularly strong positions for or against MCoC, and some may have declined involvement because of polarisation on this issue in the NHS.

We conducted semi‐structured interviews online between August 2023 and November 2024. Although different interview guides were used for different professional roles, questions generally covered the same ground and were phrased neutrally. Interview guides are available in a repository (Milton 2024). Written consent was obtained and participants could end interviews at any time. Interviews were professionally transcribed and pseudonymised. Data analysis was led by A.M. using NVivo, with double coding of selected transcripts by H.S. A.M. identifies as a feminist researcher with a background in social science, interested in power and its sociocultural expression. The coding framework was developed iteratively using an approach informed by abductive analysis (Atkinson 2018; Timmermans and Tavory 2012), drawing on a literature review in the field conducted by the authors (Middlemiss, Channon, et al. 2024), our knowledge of the CFIR (Damschroder et al. 2022) and repeated reading of transcripts. Discussions were held throughout analysis and writing with the whole research team and the PAG, as well as elements of the analysis being informally discussed with NHS staff in other parts of the research, for example, at the trust level. Further details on methods are available in the study protocol (Milton et al. 2025).

For the purposes of this paper, we brought together codes containing data about sociocultural and personal values in relation to the motivation to implement MCoC, and about evidence and advantage claims related to the CFIR innovation domain and MCoC as a ‘“thing” being implemented’ (Damschroder et al. 2022). We interpreted these through a Foucauldian framework paying attention to discursive regularities and regimes of truth and morality (Foucault 2002; Qvarsebo 2019; Rose 1999). Other themes from the overall analysis, including the macro level context in which MCoC was implemented and the national implementation process will be reported separately.

## 3 Findings

Our findings show how the implementation of MCoC in England interacts with professional and personal values, ethics and morals in the accounts of those responsible for driving change. Analysis of interviews with stakeholders in the implementation of MCoC as defined by Better Births found specific and strongly held positions on the nature of the intervention, its value and moral status, how this should be communicated to others, particularly through the use of evidence, and the characteristics of supporters and dissenters. These positions combine with epistemological positions on truth and evidence because of the need to persuade others to participate in implementation. As a result, passionate dissemination of the model is embedded into practices of MCoC implementation. The findings show how this shared vision is represented by stakeholders. We develop this in the discussion to explain the moral regime which pertains to the implementation of MCoC.

### 3.1 MCoC as the Ideal Form of Care

Midwifery continuity of carer was characterised by most of our interviewees as the quintessential form of care delivery:

We've really focused on continuity of carer because we believe, and all my team believe, that it's the one thing that can really make a difference to women and their families and their outcomes, and experiences as well.

(SH09)

Supporters of MCoC described it as the solution to multiple different issues in maternity care, including maternity safety and quality, women's autonomy and choice, personalised care, reduction in birth trauma, overmedicalisation, health outcomes for babies and health inequality. MCoC was also positioned as an overarching solution to safety concerns after a series of reports into maternity safety failures in the English NHS (Kirkup 2015; Ockenden 2020; Kirkup 2022). At times, MCoC was invoked as the solution for *every* problem in maternity service delivery, such as in this account of a regional meeting:

A midwife who's in my region was asking about how is everybody dealing with phone calls to triage and have you got a guideline. So, like, I immediately put my hand up […] and said ‘well, if you implemented continuity of carer models then you wouldn't have the issue because the woman would know who to phone and she'd probably phone earlier’. […]

And then we had a conversation about induction of labour, about the rates of induction of labour and again we had a conversation about, actually, if we did continuity of carer then we probably wouldn't have the rates of induction that we have because women will feel more supported. […]

So you've got something that will fundamentally sort everything out, so it'll sort that out, it'll sort triage out, it'll sort incidents, personalisation, it sorts everything out, changing the model.

(SH09)

MCoC was argued to be a comprehensive solution on a national scale akin to the major public health intervention of vaccination, where not using the intervention would be nonsensical:

You can have a return on investment by implementing continuity of carer, in terms of saving lives and improving outcomes and experiences. […] It's somewhat of a no brainer. […] This is like having a magic formula, this is like having the vaccine for the coronavirus, within a few weeks of the Coronavirus appearing, and not using that vaccine.

(SH15)

The importance and value of MCoC was considered extremely strong and far reaching by many participants, particularly those with a midwifery background and those who were directly involved in MCoC implementation.

### 3.2 The ‘Gold Standard’: Rhetorics of Value, Truth and Morality Around MCoC

The interviewee who characterised MCoC as the ‘magic formula’ also repeatedly used the phrase ‘gold standard’ to refer to midwifery continuity of carer. Many of our interviewees used this term spontaneously in interviews in the idiomatic sense of ‘best of the best’:

If you know absolutely, you can evidence better outcomes, that why would you … why would you go bronze standard when you can have gold?

(SH22)

The phrase was used to argue for an eventual ideal maternity service involving access to MCoC for all women, or to argue that at least MCoC should be offered to the most vulnerable. ‘Gold standard’ implicitly referenced the claims of standardised evidence‐based medicine especially as represented by evidence from large scale trials and data such as Cochrane reviews (Timmermans and Berg 2003). Using the term ‘gold standard’ about MCoC was therefore a claim about efficacy and value connecting it to the dominant values of medicine, themselves sometimes socially and politically positioned as more authoritative than the values of midwifery (McIntosh 2012). Tensions between the professions of midwifery and medicine have been repeatedly noted (e.g., McIntosh 2012; Sandall 1996; Sonmezer 2020), as has the legitimation of midwifery through connections to medicine (Foley and Faircloth 2003). Discursively connecting medicine's values to those of midwifery and MCoC by referencing a ‘gold standard’ was a way of rhetorically promoting the model of care.

The ‘gold standard’ term also connected to its original meaning standardising currency based on gold bullion: guaranteeing one truth or value by reference to another, ultimate, value. The phrase claimed the Better Births version of MCoC as an ideal form of continuity, a more ‘true’ and genuinely valuable version than other versions, involving on call availability for intrapartum care by the same midwife who provided antenatal and postnatal care:

That pure continuity model is the model that I feel best works with how people … with giving that continuity in that *real* way. […] I find that pure model of continuity, for me is the only one that can really work.

(SH01)

The ‘pure’ version of MCoC consisted of relational continuity for as many women as possible, across the whole spectrum of maternity care. Accordingly, the provision of antenatal and postnatal continuity of carer alone, as prioritised in some NHS trusts, was considered inadequate. Where provision excluded intrapartum care it failed to meet the ‘gold standard’ and thus the standard of ‘true’ continuity which could only be provided by the Better Births form of MCoC.

Ideas of truth, purity and there being an ideal or best way to provide maternity care were prevalent in the accounts of MCoC implementation. These concepts were supported by the use of other forms of moral and value‐led reasoning in discussing MCoC implementation:

For me, my narrative is that it's unethical not to do it, so we're doing women and their families a disservice because we know that it makes a difference but we're saying that you can't have it because midwives don't want to do it and midwives are supposed to be caring for women and families and using the best evidence‐based information that they've got to deliver care.

(SH09)

Particular emphasis was placed on some groups being especially in need of MCoC and therefore the moral imperative to provide it being particularly strong. Black and minority ethnic groups and women from deprived communities experience worse maternity care and outcomes than other groups (Draper et al. 2023; Office for National Statistics (ONS) 2023; Peter and Wheeler 2022) and MCoC was considered a solution to this inequality: ‘… it is an absolute responsibility we have to provide this gold standard at least to that group of women’. (SH08).

Other moral ideas about inequality and inequity were centred around the need to combat perceived discrimination based on sex:

This has got something to do with gender, I believe, there's a disparity here, because if this was something that would help men, then we might be further on, the dial may have moved in a different way. So, I think, are we serious about improving maternity outcomes and experiences or not? If we are, we need to find a way to provide the very best, for women, because that's what they deserve.

(SH15)

This participant connected poor outcomes in maternity care to sexism and the deprioritisation of women and children's needs in wider society, and presented MCoC as a way to redress this balance. Others linked a moral obligation to provide MCoC to the deservingness of pregnant women:

We did an engagement event and there was an obstetrician there, and this senior obstetrician said, ‘oh let's face it we just might as well forget about continuity of carer because the horse has bolted, so we might as well forget about it’. […] and then he said, ‘we absolutely know it's the gold standard’ […] At which point, someone in the room said ‘do you not believe women are entitled to gold standard care’?

(SH18)

Moral principles were explicitly invoked in justifying rhetorical arguments and discourses in favour of the implementation of ‘true’ MCoC provision. This included arguing that not implementing true MCoC could undermine a range of just causes, such as redressing gender inequality, alongside arguments about the deservingness of those perceived to be most vulnerable, including deprived women and black and ethnic minority women. MCoC was seen as a mechanism which, if implemented, could right a range of moral wrongs.

### 3.3 Persuasion Techniques and the Use of Evidence in MCoC Implementation

As well as the need to appeal to principles and morality in justifying the implementation of MCoC, our interviews showed that persuasion and conversion of others through argument was required to ensure implementation in different settings:

It's like a snowball, if you could just get the first few folk who are really on that, you know, on that winning … and then be able to demonstrate and demonstrate and demonstrate … that you get to that tipping point of my goodness, we *have* to do this.

(SH08)

Arguments put forward by those seeking to implement MCoC were frequently based on advantage claims for the model of care supported by understandings of clinical outcomes, many of which echoed the findings of the 2016 Cochrane review of the advantages of MCoC models of care, namely a reduced risk of pre‐term birth, neonatal death and pre‐24 weeks foetal loss, and a reduction in the use of epidural analgesia and instrumental vaginal birth (Sandall et al. 2016). This review has recently been updated and now suggests in relation to these claims there is little or no difference in pre‐term birth, uncertainty about effects of foetal loss and neonatal death, uncertainty about regional analgesia, and only a ‘likely reduction’ in instrumental vaginal birth (Sandall et al. 2024). However, much of the timescale of the Better Births MCoC implementation process was prior to publication of this revision, and the national NHS England team strongly promoted the 2016 Cochrane evidence as a clinical outcomes‐based reason for implementing the model of care, alongside evidence from Better Births about women wanting this form of care. Stakeholders who supported MCoC implementation invoked the review to persuade others to provide the ‘best’ model of care for women, evidenced by the ‘best’ evidence. Cochrane review evidence was considered extremely strong, despite the limitations of this form of evidence in healthcare interventions (Howick et al. 2022). Sometimes evidence from the 2016 review was set seamlessly alongside other advantage claims which that review did not evidence, such as reduced rates of induction or caesarean birth:

Somebody was asking me yesterday, what are one of the things that you could suggest would help with the increase in requests for elective caesareans. I said continuity, because sometimes that's just born out of fear. But if you've got continuity and got that relationship and you know who's going to be at your birth and you know you're going to get the support, you're more likely to do that [vaginal birth]. So rather than say ‘oh, there's so many sections, we won't do it’, let's do it, so that we get less sections.

(SH10)

Claims such as these were based in practice and rested on inferences about the potential mechanisms through which MCoC might produce different outcomes for women. Arguments implicitly positioning some kinds of birth as preferable to others, and more achievable within the MCoC model of care, also aligned the model with demedicalised, midwife‐led forms of birth. As with the overarching claims for the value of MCoC as a solution to multiple maternity service problems described above, evidence claims were used by some participants to expand the reach of MCoC beyond outcomes for, and care of, individual women and babies and into public health and the functioning of NHS institutions. For example, they referred to wider public health outcomes such as decreased smoking in pregnant women and improved breastfeeding rates, or cost‐related claims such as women being discharged quicker after birth. Such advantage claims were passionately espoused by supportive participants:

I mean some of the most powerful presentations I've been shown, when I've gone round and done visits in response to the continuity of carer is the number of premature births have dropped right down … all sorts of things, inductions have dropped down, the number of straightforward births without interventions have increased, it's amazing this relational trust.

(SH08)

The origins of the evidence invoked to persuade about the value of MCoC not only included the 2016 Cochrane systematic review but also additional local evidence generated within specific implementation localities, such as internal staff surveys or changes in intervention rates in specific care teams over relatively short periods of time. Distinctions between the origin, type, quality and scope of ‘the evidence’ were not critically appraised or discussed when invoked in support for MCoC implementation, but locally based evidence perceived to be on the ‘other side’, being unsupportive of, or oppositional to, MCoC and its implementation, was critiqued and dismissed:

The DoMs and HoMs [Directors and Heads of Midwifery] all felt that it was too difficult to do ‐ erm some of them do wanna do it and can see the benefits, but a lot of them were saying ‘well I've done a survey and the midwives don't want to do it’. So *we're* saying ‘well *we've* done a workshop and they *do* wanna do it’ so yeah, somebody's not telling the truth here, are they, you know what I mean?

(SH09)

Put head‐to‐head, evidence perceived to be against the implementation of MCoC was represented as inadequate by those invested in its implementation. Interventions limiting the policy were also critiqued, for example, some participants rejected the Independent Maternity Review recommendations limiting MCoC rollout (Ockenden 2022) because the NHS Trust where the review took place did not have MCoC at the time, so they considered the findings to be overreaching the review's evidence base. Some participants also queried the expertise of the Independent Maternity Review's author Donna Ockenden, or made claims about the lack of evidence base for other maternity interventions or models of care, arguing that since evidence was not needed in those cases, a stricter standard should not be required of MCoC. This shifting of goal posts and selection of different arguments and evidence to support MCoC depending on context was critiqued by one more dissenting nonmidwifery stakeholder:

There's this general sense that we should be supportive of Continuity of Carer because it's ‘a good thing’. […] People think … have a general sense that continuity is a good thing to do and will pick and choose the evidence that supports it, depending on what the evidence says. […] That's played out a little bit in response to the most recent updated Cochrane Review. Which is, felt a little bit like, ‘But don’t worry, we should continue doing it anyway because there's always other good things that might happen’.

(SH31)

The moral imperative of implementing MCoC as a model of care can require marshalling evidence and aligning multiple arguments to support the policy as the ‘right’ thing to do.

### 3.4 Dissent and Conversion

At the same time, lack of knowledge and understanding of ‘the evidence’ and its support for implementation was cited by both national and regional stakeholders as a reason why MCoC implementation was not adopted wholeheartedly within maternity services. Dissent was presented as a lack of faith in the MCoC model of care due to inexperience, allied to a deficit of knowledge and confidence, resulting in local implementation difficulties:

Where I've seen it fail is where the senior midwifery leadership don't believe in it, sometimes until you've worked in it and seen it work well it's hard to believe. You do continue to believe these rumours, you believe that you need more staff, and actually you don't if you've got your establishment right you can move that. So, again it's I think having the knowledge and confidence really knowing how to work your establishments.

(SH11)

Sometimes dissenters could be ‘finally’ converted by persuasion and the clarity of those in the know:

A really good example was one Director of Midwifery, we know she can't stand continuity, absolutely, she does not like it, and has always not engaged with our continuity of carer midwives and all the rest of it, but I think because we had these honest, open conversations about you know, what we're gonna do in the future, and what do they want to do and all the rest of it, she's finally engaged, and she reached out and contacted the Continuity Carer Lead to say, ‘Can we talk about this?’ Cos it was almost like, for the first time, there was honesty in the room.

(SH14)

The positioning of dissent as coming from ignorance, unwillingness or lack of faith sat alongside the positioning of noninvolvement as morally questionable and served to other those NHS staff who expressed reservations about aspects of the MCoC policy:

There were a number of midwifery leaders […] who stood up and said ‘I can't do this, it's unsafe’. They got branded then as a poor leader. […] Nobody dared to say anything about midwifery continuity, and then the positiveness and the happy clappiness just continued, so it gave such a false impression of what was really happening.

(SH05)

Personally what I saw was that you couldn't have a conversation with particular individuals because it's all about that one mission, nothing else mattered.

(SH06)

Stakeholders within the NHS who acknowledged the difficulties of implementing MCoC were represented as, or represented themselves as, sceptical of the process of implementation rather than the principles behind MCoC:

In heart I believe it probably is the right thing to do, but the *how* it's implemented and *when* it's implemented I have such huge concerns about.

(SH05)

Critique of MCoC implementation could only extend so far for those participants we interviewed who were invested to some degree in the policy implementation.

### 3.5 Passion, Vocation and Midwifery Values

For many of our participants, the core incentive for implementing MCoC aligned with the relational, ‘with‐woman’ professional values of midwifery rather than the clinical effectiveness reported in the 2016 Cochrane review. Continuity reached back into the vocational history and professional origins of midwifery:

Historically, that's how midwives worked. You know, my granny got somebody to knock on the midwife's door and the midwife came round and delivered the baby.

(SH24)

We had quite a few younger midwives who are quite keen to work in that way and I think they'd gone into their training sort of hearing about continuity and the benefits of it and, you know, watched *Call The Midwife* and they were like, ‘oh that's what we should be doing’.

(SH23)

Participants described how MCoC was related to the fundamental principles of midwifery: ‘how midwifery is intended to be’ (SH32). The MCoC model of care was aligned in this way with true and pure moral values. Willingness to participate in the model was central to midwifery values and espoused by ‘truly women‐centred’ midwives who ‘really want to make a difference and give themselves freely to make that difference’ (SH12). Several participants who felt particularly strongly about the moral value of MCoC identified what they considered to be a problematic shift in values in the current workforce, who were less inclined to work in a continuity model:

I think midwives are sold on why continuity is good, but I think the problem is that nowadays […] colleagues are really hot on things like their own wellbeing, getting a work‐life balance and so that is a big change that I've seen from 30 years ago, when you came into midwifery and you basically did what you were told.

(SH11)

Similarly, a lack of willingness to participate in MCoC was represented as lack of vocational commitment. Participants from within midwifery and in other stakeholder groups who supported the implementation of MCoC explicitly linked some midwives' reluctance to work in MCoC models to a lack of personal commitment to midwifery and woman‐centred care:

I think we've kind of, lost our way a little bit, in terms of why we're really here. And my view is that we're here to serve women and babies and their families. Now some people don't like the word, ‘serve’, you know, we're here to ‘provide care’ for women, babies and their families.

(SH15)

For this participant, the very existence and purpose of midwifery was best understood as a service role, traditionally involving deep personal commitment, contrasted with an alternative vision of a less committed abstract ‘provision’ of care, a theme echoed by others:

I do question sometimes why some people have become a midwife. Or I ask them to reflect and remember and remind themselves why they did become a midwife.

(SH12)

Participants such as these, who strongly promoted the importance of MCoC implementation as a moral task, could be dismissive of the professional values of others who questioned MCoC. They expressed a sense of struggling against the odds, a moral task, a ‘vision’ in which ‘moral injury’ (SH08) was experienced when there was pushback during implementation. Great effort was needed to implement change, including the involvement of ‘prophetic’ leadership in MCoC implementation (SH08), and ‘unique’ and ‘courageous’ individuals (SH08):

It was a real beast, and when it was going well, it was brilliant, you know, I was really committed and passionate about it and was quite happy to work sixty‐hour weeks, so that it was done, you know, and that it was working well. Because it felt really important and massive, you know, it just felt like such an amazing thing to achieve.

(SH34)

By contrast, those who were critical of MCoC were represented as weak, making ‘excuses’ (SH08) for why they could not implement the model, of using staffing issues as a dubious reason for deprioritising implementation, ‘just giving up’ (SH11), being ‘naysayers’ (SH08, SH15) and putting it in the ‘too difficult pile’ (SH21). This interpretation had consequences for those who were seeking to implement MCoC but not succeeding, who were then implicitly aligned with irrational ‘naysayers’ who did not understand, or would not engage with, the model of care represented as so crucial for pregnant and birthing women. Asked whether it was difficult for staff to say no to working in MCoC, a regional manager told us:

It almost feels like it's weaponised that passion that staff have. Because when you talk to all the staff, they do say, you know, 'we want to support them, we want to advocate for them, we want them to have the best outcomes, we want them to have a good experience. We love seeing them from booking to outcome at the end’. And it all sounds very nice, until you dump it in the context that maternity services are currently in. And I think that's where a lot of the moral injury for the staff is, is really coming in.

(SH28)

Some stakeholders noted the degree to which passion and commitment were necessary for the implementation to happen at all in the context of limited service and organisational support for the changes:

I'm not an expert in project management, I'm not an expert in organisational transformation, or change management. I was a consultant midwife, reasonably good at what I did, but then this landed on me, without proper preparation, without proper support. And we did what we could […] We did what we could with the resources we had. And I think that it ran on goodwill. Rather than on expertise or resource.

(SH35)

For this participant, the NHS relied on a ‘discretionary effort’ (SH35), from the midwifery workforce based on emotional and vocational commitment to the NHS as a moral good, in a context of limited financial and institutional support for its implementation.

## 4 Discussion: The Moral Regime of MCoC Implementation

The MCoC model of care, as the ‘thing’ to be implemented (Damschroder et al. 2022), is understood by of our national and regional interviewees as imbued with intrinsic and special value. Many of our participants reify MCoC as ‘the magic formula’, the ‘gold standard’ and ‘the best’. It is aligned with morally‐loaded principles of purity, truth, reality, and intrinsic rightness. Women who are the object of the care model ‘deserve’ this level of care, and midwives should ‘believe’ in it, because it is a ‘natural fit’ with their other professional concerns. The implementation of this model of care is represented as fundamentally ethical, a ‘duty’ or ‘responsibility’ connected to vocation and service and underpinned by moral principles.

When the moral positioning of MCoC as a model of care is combined with midwifery professional and personal values of providing the best care for women, it can become imperative to participate in the implementation of MCoC. The individual midwife‐subject has a vocational responsibility enjoining her to participate, to dedicate great personal efforts, and to spread the word to others in a form of evangelism. Moral regimes are aligned with belief, idealism and passion, and in the case of MCoC implementation the ethics of care centres on the deservingness of women, construed as vulnerable and in need of the midwife‐subject's agency.

In a narrative review of MCoC implementation we found midwifery professional values are closely aligned to the values embedded in MCoC (Middlemiss, Channon, et al. 2024), and the personal commitment required of midwives in Better Births MCoC has been noted elsewhere (Madsen 2025). Our empirical work supports these findings. When core values found in the midwifery profession in support of MCoC are also the personal values of individual midwives, and are supported by available external evidence, then the possibilities of what can be said (Rose 1999) are both strengthened and constrained by the moral regime (Qvarsebo 2019), which privileges some positions on MCoC and excludes others. Participation in MCoC thus produces the individual midwife‐subject as a moral being, and can situate those who critique, or are perceived as less invested, as irrational ‘naysayers’, lacking in understanding of evidence and in need of persuasion by any means to bring them to the correct way of thinking. Dissent becomes difficult, and those who do not comply with the moral regime are othered, with the potential for emotionally charged conflict. The consequences of a moral and values driven approach to policy implementation is that any implementation failure looks like a moral failure for the individuals concerned, striking at their sense of themselves as moral and ethical subjects. They may, as one participant described, work 60‐h weeks and be the person to whom all queries and problems are directed, but if the implementation is not successful then this may be construed as the failure of an individual, rather than that of a model of care, the flawed collective implementation of a policy, or the limitations of a healthcare system.

### 4.1 Evangelism and the Invoking of ‘the Evidence’

Others have situated moral regimes purely within ethical politics (Qvarsebo 2019), but we argue that in the case of MCoC implementation, ethical governmentality is entwined with a truth regime which relies on epistemological validity and the role of persuasion of others, consistent with acknowledgement that evidence in policy is politically and culturally situated (Boaz et al. 2019; Cairney 2019). Dixon‐Woods et al. (2013) identified ‘comfort‐seeking behaviours’ in the use of evidence in attempts to improve patient safety in the NHS, whereby data was sought in a way which aimed to reassure management and senior teams and in which concerned or critical comments were dismissed as disruptive.

The moral regime of MCoC has similar features in terms of the lack of attention to different types and weights of evidence. It is likely that there was little incentive for individuals to engage in detail with evidence for a policy which was passed to them in a hierarchical NHS system. As a consequence, evidence for the use of MCoC was routinely presented to us by our participants as monolithic and of equal validity, with peer‐reviewed outcomes of MCoC from the 2016 Cochrane review (Sandall et al. 2016) being set alongside small scale, local, time‐bound evidence‐gathering. There was a degree of vagueness in the presentation of ‘the evidence’, and it was sometimes presented as the answer to *all* problems in maternity services. We have argued elsewhere (Middlemiss, Channon, et al. 2024) that research which is positively biased towards MCoC as a form of care has a tendency to select evidence which is supportive of the model. In the interviews reported here, we also find a similar tendency to selectively use evidence to advance the case for implementation of MCoC. This is consistent with the political and instrumental use of evidence in policymaking (Boaz et al. 2019; Cairney 2019). Furthermore, those who questioned or did not accept ‘the evidence’ were sometimes presented as either ignorant or wilful, echoing the manner in which challenge was dismissed in Dixon‐Woods et al.'s (2013) analysis of comfort‐seeking data‐use behaviours in the NHS.

We contend that ‘the evidence’ in MCoC implementation is a regime of truth, defining what is counted as true, who can produce this truth, and the political consequences of speaking (or not speaking) a truth (Foucault 2003b; Rose 1999). However, this regime of truth is intertwined with the moral regime of MCoC implementation. The reason ‘the evidence’ is so important during implementation is because change to the new model is morally required, necessitating action such as the conversion of ‘naysayers’ and the personal commitment of the moral midwife‐subject. One route to conversion is the presentation of evidence as truth, which is political and symbolic in its justification of preexisting preferences (Weiss et al. 2016). This theoretical connection between evidence and values, and personal and professional motivation and subjectivity, adds a new dimension to attempts to understand implementation of policy such as MCoC in maternity services.

## 5 Conclusion

In this paper, we have described a moral regime found in the implementation of the maternity reform policy of MCoC which is coterminous with core values in midwifery as a profession and also with the values of many individual midwives, producing a midwife subjectivity which has ethical grounding. The moral regime is intertwined with a regime of truth around the use of evidence to persuade others to engage with implementation. Our overarching research project seeks to identify barriers to, and facilitators of, the implementation of MCoC in the English NHS and we argue that the moral regime of MCoC is both a facilitator and a barrier in the context of this centrally driven and evaluated policy implementation. The regime provides authority for attempts to implement MCoC, and as such may act as a facilitator of implementation, drawing individuals and institutions into these efforts. Yet, the evangelist passion with which MCoC is promoted may be alienating to those whose values are not exactly aligned with the model of care, or who critique the implementation. The moral regime may then act as a barrier to engagement with implementation. The production of midwife subjectivities within the moral regime may locate failure of implementation projects in individual or moral failure rather than complex systemic processes. Furthermore, the moral regime of MCoC implementation which drives the need for change may lead adherents to engage less critically with evidence claims around the model and its claims to truth, which may impede implementation in the longer term if those evidence claims are poorly founded, or perceived to be, or if they change over time.

Although some of the values embedded in the MCoC moral regime are specific to the fit between this policy and midwifery's ‘with‐woman’ relational ethos, the concept of a moral regime, its relationship to evidence, and its role in implementing health systems change is likely to be applicable more broadly. For example, principles of relational care underpin other roles in healthcare such as general practitioner service delivery in the NHS (Fox et al. 2024; Leach et al. 2025). Our findings also contribute to general theoretical understandings of the operation of power in national healthcare systems. We have shown how moral values not only underpin health systems (Bartosch et al. 2024) but are agentially used during change processes. Moral regimes are not static, but are dynamic, active, and contested political processes within health systems. They can be brought alongside truth regimes to strengthen arguments in the politics of implementation of healthcare improvement. A strong, internally coherent moral regime can be employed in seeking to motivate behaviour in healthcare settings. However, such a moral regime produces further political ramifications when counter arguments become unsayable in a tightly prescribed moral regime coterminous with a truth regime. Tracing moral regimes in healthcare alongside truth regimes can offer insight into the role of sociocultural values in the politics of healthcare, and the possibilities and limitations of agency in healthcare systems.

## Author Contributions

**Aimee Louise Middlemiss:** conceptualization (lead), formal analysis (lead), investigation (equal), methodology (equal), writing – original draft (lead), writing – review and editing (equal). **Susan Channon:** funding acquisition (equal), investigation (equal), methodology (equal), writing – review and editing (equal). **Julia Sanders:** funding acquisition (equal), writing – review and editing (equal). **Heather Strange:** formal analysis (supporting), investigation (equal), writing – review and editing (equal). **Rebecca Milton:** funding acquisition (equal), methodology (supporting), writing – review and editing (equal). **Sara Kenyon:** funding acquisition (equal), writing – review and editing (equal). **Tina Prendeville:** writing – review and editing (equal). **Susan Barry:** writing – review and editing (equal). **Aled Jones:** conceptualization (supporting), funding acquisition (lead), investigation (equal), methodology (equal), writing – review and editing (equal).

## Ethics Statement

Ethical approval was obtained from the University of Plymouth project reference number 3906.

## Consent

Consent was obtained in electronic written form from all participants.

## Conflicts of Interest

The authors declare no conflicts of interest.

## Permission to Reproduce Material From Other Sources

The authors have nothing to report.

## Data Availability

The authors have nothing to report.

## Endnotes

^1^
We use the terms ‘woman’ and ‘baby’ in describing the caring activities of midwives because of the historical resonance with the profession of midwifery, but we note that not all foetuses will become babies, and that not all birthing people identify as women.
